# Multifocal Osteoblastoma of the Jaws: A Very Rare Case Report

**DOI:** 10.30476/DENTJODS.2021.90454.1491

**Published:** 2022-09

**Authors:** Mohammad Moshref, Alireza Modarresi, Negar Razzaghi

**Affiliations:** 1 Retired Professor of Stomatology and Oral & Maxillofacial Pathology of Shaheed Beheshti University of Medical Sciences, Tehran, Iran; 2 Dept. of Oral and Maxillofacial Surgery, Craniomaxillofacial Research Center, Tehran Islamic Azad University of Medical Sciences, Dental Branch, Tehran, Iran; 3 Member of Craniomaxillofacial Research Center, Tehran Islamic Azad University of Medical Sciences, Dental Branch, Tehran, Iran

**Keywords:** Multifocal, Osteoblastoma, Jaws, Enucleation, Peripheral ostectomy

## Abstract

Osteoblastoma is a solitary benign bone-forming neoplasm, which comprises 1% of all primary bone tumors. Multifocal benign osteoblastoma of the jaws is very rare. Osteoblastoma must be differentiated from other similar bone-forming lesions such as osteoid osteoma and osteosarcoma for correct diagnosis and proper treatment planning. Therefore, precise examination of the patient and correlation with radiographic and histological features are essential for the best treatment and prognosis. This study reports a rare case of multifocal osteoblastoma in a 30-year-old female, involving the mandible and the maxilla, which was treated by surgical excision, iliac bone graft reconstruction, and implantation. Complete surgical excision is necessary to treat osteoblastoma with a good prognosis. The patient was followed-up for four years postoperatively, and there were no signs of recurrence in the panoramic view or the clinical examination

## Introduction

Osteoblastoma (OB) is a solitary benign tumor that was first described by Jaffe and Lichtenstein in 1956 [ 1 - 2 ]. OB occurs during the second decade of life with a male predilection (sex ratio of 2:1) [ 3 - 5 ]. 

This benign bone-forming neoplasm is uncommon and comprises 1% of all primary bone tumors and only 3.5% of all benign bone tumors [ 6 - 7 ]. It is most commonly found in the vertebral column and the long bone-s. About 10%-12% of OBs occur within the gnathic bone with the mandibular posterior region being affected 2-3 times more frequently than the maxilla [ 2 , 4 - 6 ]. Patients often present with a painful mass not relieved by the non-steroidal anti-inflammatory drugs (NSAIDs) and with clinical signs related to swelling, obstruction, tooth mobility, tooth displacement, root resorption or compression of the surrounding structures [ 3 , 8 - 9 ]. Rarely, these tumors may exhibit progressive growth, local recurrence, and malignant transformation [ 2 ]. Aggressive osteoblastoma (AO) is the type of osteoblastoma, which is clinically and radiographically larger (˃4 cm) than conventional OB [ 10 ]. AO affects an older age group and pain is common. The probability of recurrence is 13.6% for conventional OB and 50% for AO [ 11 ].

OB is a rare bone tumor, which is usually seen as solitary. In very rare cases, it may occur as an isolated focus in more than one bone or as multiple foci within one or more bones [ 10 , 12 ].

To date, only one case of multifocal OB of the maxilla and the mandible has been reported [ 13 ]. 

This study reports a rare case of multifocal OB in a 30-year-old female, involving the mandible and the maxilla, which was treated by surgical excision iliac bone graft reconstruction and implantation. 

## Case Presentation

A 30-year-old woman with mild pain and swelling on the right side of the mandible since two months ago referred to the dentist. She had taken NSAID to relief the mild pain. Panoramic radiography was taken. The lower right first molar was extracted due to luxation. No other treatment was performed. She was referred to an oral and maxillofacial surgeon. There was no history of trauma to the maxillofacial region. The patient had a history of hypothyroidism for the last seven years and was taking levothyroxine (10 mg per day). 

In the clinical examination, mild extraoral swelling on the buccal aspect of the right body of the mandible and intraoral moderate swelling from the right first premolar to the right retromolar region of the mandible were found. The clinical differential diagnoses of this lesion were residual cyst, keratocystic odontogenic tumor (KOT), ameloblastoma, and odontogenic myxoma. The swelling was firm with significant buccal and lingual expansion of the cortical bone. No neurosensory disturbance or trismus was found. The texture and the color of the overlying mucosa and the skin were normal.

Clinically, there was no abnormality in the left posterior mandible or the anterior maxillary region. 

**Figure 1:**
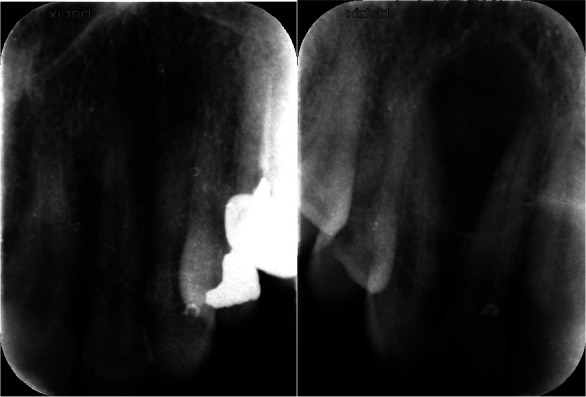
Pre-operative panoramic view, arrows show multifocal lesion. Periapical view of maxilla lesion

Radiolucent, well-defined, partially corticated lesions were seen on the panoramic radiograph, located on the right side of the mandible with the dimensions of 40×30×20mm , close to the lower border, from the distal aspect of the right first premolar to the distal aspect of the right second molar. Lesions were also seen on the left side of the mandible around the apex of the third molar and in the maxilla between the roots of the lateral incisor and the canine on both sides (Figure 1). Radiographic differential diagnosis of right mandibular lesion was radicular cyst, KOT, central giant cell granuloma (CGCG), unicystic ameloblastoma, odontogenic myxoma, and osteoblastoma. Radiological differential diagnosis of left mandibular lesion was radicular cyst and KOT. Radiological differential diagnosis of maxillary lesions was lateral periodontal cyst, KOT and radicular cyst.

**Figure 2:**
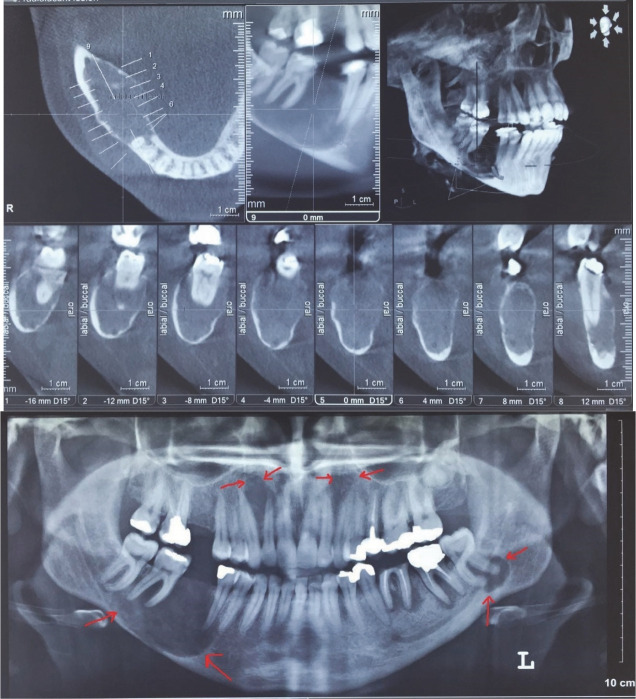
Pre-operative cone-beam computed tomography (CBCT) view, buccal and lingual bone thinning, and perforation, push the inferior alveolar nerve toward the lower border

 The lesions of the maxilla and the left mandible were smaller than the lesions on the right mandible. In the cone-beam computed tomography (CBCT) images, fine bony septa were seen extending from the outside into the lesion. Loss of the lamina dura was detected at the distal aspect of the lower right second premolar with resorption of the lower right first premolar root. The lesion pushed the inferior alveolar nerve towards the lower border of the mandible, causing lingual bone thinning (Figure 2). 

**Figure 3:**
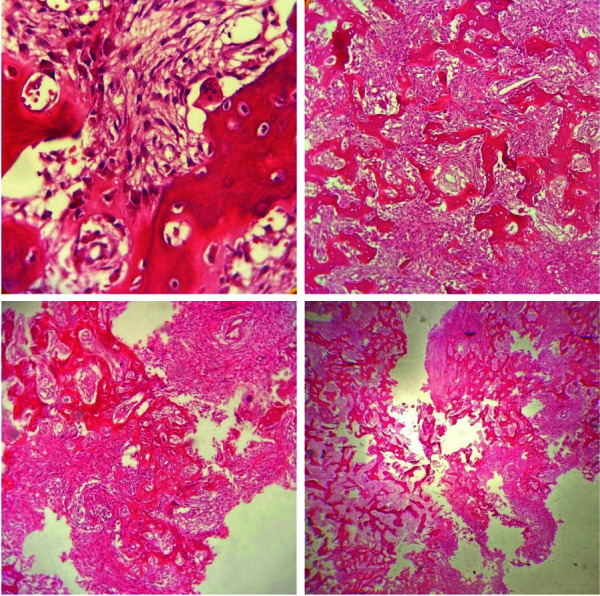
Histopathological images showed stroma containing osteoblasts, scattered dilated vascular channels, and large sheets of irregular trabeculae in osteoid material rimmed by osteoblast with ample cytoplasm and hyperchromatic nuclei

Bone scintigraphy demonstrated bilaterally increased tracer accumulation (hot spot) at the maxilla and the mandible. These findings show areas where the bone is breaking down. At first, the left side of the mandible was operated, and then the lower left first, second and third molars were extracted. Then, an excisional biopsy was performed. An incisional biopsy was also performed on the right body of the mandible under local anesthesia. The histopathological assessment showed multiple pieces of a rather loose fibrous connective tissue containing scattered dilated vascular channels and large masses of osteoid mineralized material with prominent reversal lines. These large sheets of irregular trabeculae contained numerous osteoblasts with an ample cytoplasm and hyperchromatic nuclei (Figure 3). 

Scattered multinucleated osteoclast-like cells were seen at the periphery of these large masses, surrounding the trabeculae.

Focal areas of hemorrhage, a central zone of increased vascularity, and some large epithelioid osteoblasts were also evident. The histological diagnosis was benign OB. The final diagnosis of benign OB was made based on the histological, radiological, and clinical findings.

The maxillary lesions were removed during the second operation under local anesthesia with the same pathology report as the mandibular lesions.

The complete excision of the tumor mass and intraoral insertion of a reconstruction plate was planned under general anesthesia. 

**Figure 4:**
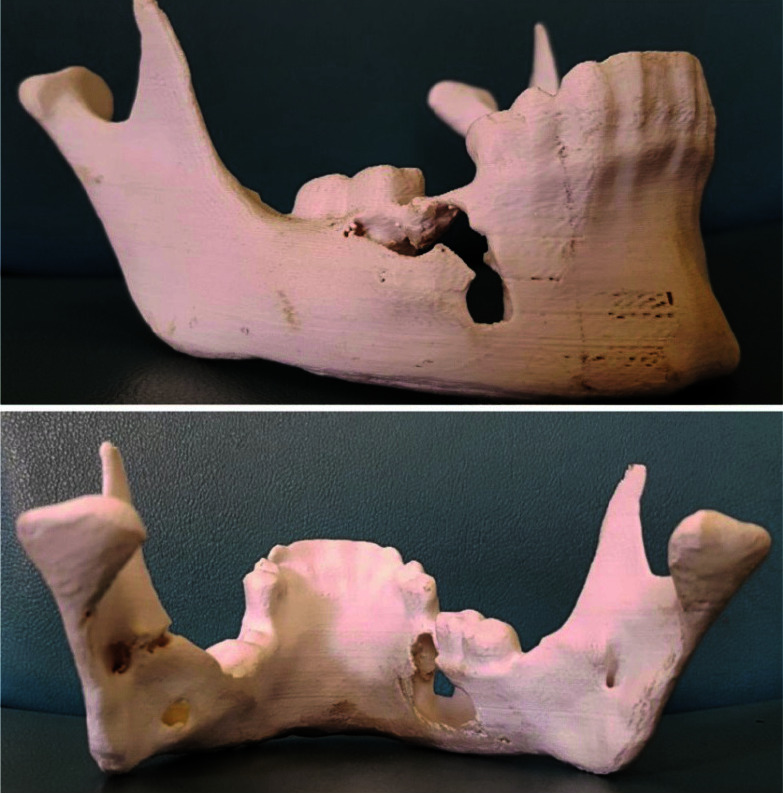
Pre-operative medical stereolithography (3D printing model of the mandible)

Computed tomographic (CT) scans of the maxillofacial skeleton were obtained and sent to the laboratory for the fabrication of a model. A plaster medical rapid prototyping model of the mandible was prepared using three-dimensional (3D) printing (Figure 4).

The reconstruction margins were designed by the surgeon and a technician bent and fitted the titanium reconstruction plate, which was sterilized by autoclaving. 

**Figure 5:**
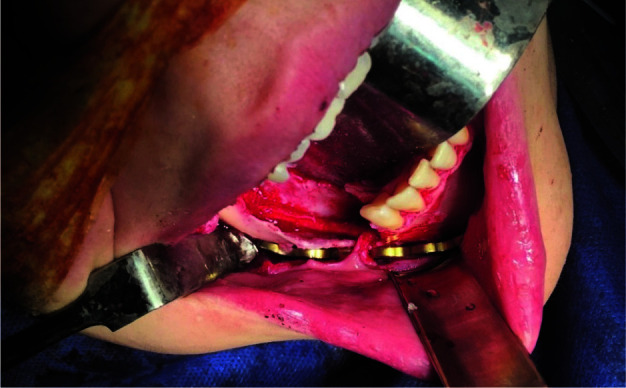
Intra-operative view, pre-bent reconstruction plate was fixed intraorally; the inferior alveolar nerve was preserved

**Figure 6:**
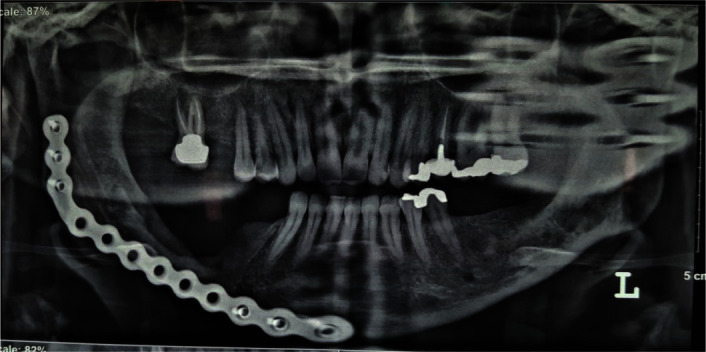
Post-operative panoramic view, 6 months after operation

The tumor mass was completely excised through a buccal vestibular incision extending from the canine to the anterior border of the ramus. The tumor was circum scribed to facilitate the excision. After the excision of the tumor, peripheral ostectomy was performed. Finally, 5mm of the inferior border of the mandible remained. Then, the pre-bent reconstruction plate was fixed intraorally to the mandible using six titanium screws. The inferior alveolar nerve was preserved with no injury (Figure 5). There were no clinical or radiographic signs of recurrence at the one-, three-, six- months and one-, two-, three-, four-years follow-ups (Figure 6).

**Figure 7:**
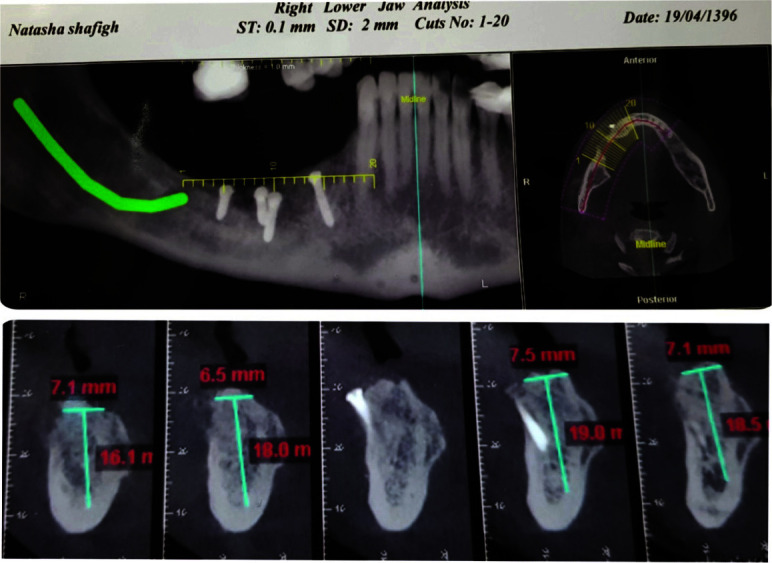
Cone-beam computed tomography (CBCT) after reconstruction with iliac bone graft

**Figure 8:**
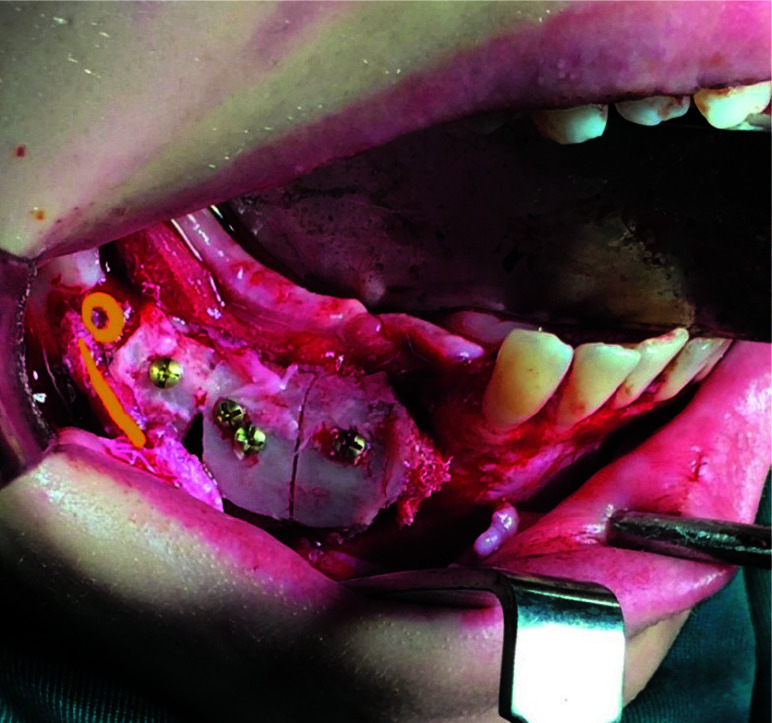
Intra-operative view (secondary surgery), iliac bone graft, the line shows inferior alveolar nerve and the circle shows new mental foramen

**Figure 9:**
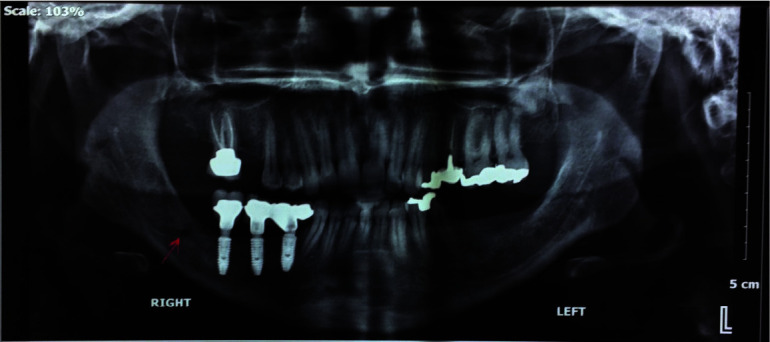
Post-operative panoramic view, reconstruction of the right mandible with implantation, arrow shows the new mental foramen position

After six months, the reconstruction plate was removed intraorally, and the hard tissue was reconstructed using an iliac bone graft under general anesthesia (Figure 7). The inferior alveolar nerve was repositioned laterally. Then, the iliac bone graft was fixed with four screws (Figure 8). There was no histological evidence of recurrence. Four months later, implantation of the right mandibular body was performed under local anesthesia (Figure 9).

## Discussion

OB is a rare bone tumor [ 3 , 6 - 7 ]. It is more frequently seen in the long bones and it rarely involves the maxillofacial region [ 1 , 4 - 6 , 11 ]. Commonly, OB is a solitary bone tumor [ 1 ], but some multiple cases have been observed in the long bones [ 12 , 14 - 15 ].

Yu *et al.* [ 12 ] described a case of an 8-year-old girl who was diagnosed with the Cushing syndrome and multiple OB in the femur, the pelvis, and the skull base. Kyriakos *et al.* [ 14 ] presented two adult patients with multifocal osteolytic lesions, radiographically simulating a vascular tumor. Multiple bones have been involved in one patient [ 20 ]. Adler [ 15 ] reported a 9-year-old child with multiple OB in multiple bones of the right hand.

To Date, there is only one report of multiple lesions in the jaws. Asada *et al.* [ 13 ] described a case of OBs at multiple sites of the maxilla and the mandible, one of which was accompanied by simple bone cysts. They presented a 38-year-old woman with painless, non- growing swelling on the right side of the mandible for three years. The tumors produced hazy or ground-glass- like semi-radiolucent lesions with well-defined or poorly dely defined margins on conventional radiographs.

The final histological diagnosis was multiple benign OB accompanied by simple bone cysts. The lesion was removed by en bloc resection followed by immediate reconstruction with the iliac bone and a metal plate. There was no sign of recurrence of the lesions one year and six months postoperatively [ 13 ]. 

Radiological manifestations of OB are variable and nonspecific but usually indicate a lesion that is generally greater than 2cm, oval, or round, expansile, well circumscribed, and predominantly lytic with a rim of reactive sclerosis. The central portion can be completely lytic but usually shows calcifications, at least focally [ 16 ]. In the present case, the lesions showed well-defined, partially corticated, and expansile borders without a sclerotic rim or foci of patchy radiopacities within the radiolucency. The radiological findings described in our case are similar to that of the benign OB cases presented in the literature considering that our case did not show intralesional calcifications. Accordingly, based on the radiological features, the differential diagnoses included residual cysts, CGCG, KOT, and ameloblastic variants.

Considering the rarity of multifocal benign OB of the jaws, there may be some differential diagnoses such as fibro-osseous lesions, osteoid osteoma (OO), osteosarcoma, cementoblastoma, and ossifying fibroma.

OB and OO are closely related benign bone tumors. Indeed, OO is the main differential diagnosis challenge of our case. The OO exhibits more limited growth potential than OB (less than 1.5-2cm). The pain of OO is relieved by the NSAIDs. Radiographically, OO manifests an intracortical nidus with different amounts of mineralization, cortical thickening, sclerosis, and bone marrow edema [ 17 ]. The surrounding reactive sclerosis is less prominent in OB than in OO. The lesions in the present case were differentiated from OO because of the tumor size on the right side of the mandible and the absence of a central nidus of osteoid tissue. 

Histologically, OB is composed of haphazardly deposited trabeculae or sheets of woven bone and osteoid rimmed by osteoblasts and scattered osteoclasts that are enmeshed in a richly vascular stroma [ 18 - 19 ]. In the present case, the microscopic assessment showed scattered dilated vascular channels and large masses of osteoid mineralized material with prominent reversal lines. These large sheets of irregular trabeculae contained numerous osteoblasts. Surrounding the trabeculae were scattered multinucleated osteoclast-like cells. Some large epithelioid osteoblasts were also evident. These epithelioid osteoblasts are one of the characteristics of AO. 

The differentiation of AO and low-grade osteosarcoma (LGO) should be considered. They are different in cells, stroma, and the matrix. AO is characterized by the presence of large epithelioid cells with an ample amount of eosinophilic cytoplasm and hyper-cellular stroma. The trabeculae are large and irregular with osteoblastic rimming and may show reversal lines in the matrix with increased mitotic activity and sheets or lace-like areas of osteoid production, but LGO is characterized by the presence of hyperchromatic nuclei. The neoplastic cells inclined to grow in an angiocentric manner. The stroma shows dens, pink, refractory, and amorphous intercellular material, known as osteoid and the matrix contains normal bone trabeculae [ 20 ]. The present lesions were distinguished from LGO because of the slow growth and the absence of LGO characteristics, such as nuclear atypia and increased mitotic activity. The present case showed large sheets of irregular trabeculae containing numerous osteoblasts with an ample cytoplasm and hyperchromatic nuclei. 

The pathology report indicated benign OB in the maxilla and the mandible. Right mandible lesion shows more epithelioid osteoblastoma cells so the diagnosis for this lesion is AO; therefore, it was named multifocal OB of the jaws. Incidentally, we can designate the lesion located on the right side of the mandible as AO, concerning its size and microscopic features. The clinical and radiographical appearance of OB is very similar to peripheral ossifying fibroma or any fibro-osseous lesion. The central histological feature to differentiate this pattern from fibro-osseous lesions is that the stroma does not consist of cellular spindle cells but rather a loose vascular stroma with numerous prominent epithelioid-type osteoblasts [ 21 ].

The common approach to treating OB is resection with a 5mm safety margin [ 2 , 7 ]. The alternative therapies of enucleation, curettage, and peripheral osteotomy are also valid but have specifically limited indications [ 4 , 20 ]. Kyriakos *et al.* [ 14 ] described two adult patients with multifocal osteolytic lesions in the leg, which were removed by en bloc excision. Mahajan *et al.* [ 4 ] described a 45-year-old female with swelling on the right lower back since five years ago, which was removed through excision.

Regarding the present case, according to the extension of the lesion and the histopathological findings, conservative treatment was chosen, including enucleation, curettage, and peripheral intraoral ostectomy. The inferior alveolar nerve was preserved and displaced laterally.

There were no clinical or radiographic signs of recurrence at the one-, three-, six- months and one-, two-, three-, four- year follow-ups. 

Written informed consent was obtained from patient prior to this case presentation.

## Conclusion

Multifocal benign OB of the jaws is a very rare bone tumor. This tumor must be differentiated from other similar bone-forming lesions such as OO and osteosarcoma for correct diagnosis and proper treatment planning. Therefore, precise examination of the patient and correlation with radiographic and histological features are essential for the best treatment and prognosis. Complete surgical excision is necessary to treat OB with a good prognosis.

## Conflict of Interests

The authors declare that they have no conflict of interest. 
